# Role of Mig-6 in adipose tissue: Implications for glucose metabolism and insulin resistance

**DOI:** 10.1371/journal.pone.0314289

**Published:** 2025-02-12

**Authors:** Ji Min Kim, Joung Youl Lim, Sorim Choung, Ok Soon Kim, Kyoung Hye Joung, Ju Hee Lee, Hyun Jin Kim, Bon Jeong Ku

**Affiliations:** 1 Department of Internal Medicine, Chungnam National University Sejong Hospital, Sejong, South Korea; 2 Department of Internal Medicine, Chungnam National University School of Medicine, Daejeon, South Korea; 3 Department of Medical Science, Chungnam National University School of Medicine, Daejeon, South Korea; Army Medical University, CHINA

## Abstract

**Background:**

Insulin resistance is a hallmark of type 2 diabetes mellitus (T2DM) and is associated with metabolic disorders. Adipose tissue plays a crucial role in regulating whole-body energy balance and glucose homeostasis. Mitogen-inducible gene 6 (Mig-6) is a negative feedback regulator of receptor tyrosine kinases, including epidermal growth factor receptor (EGFR). This study aims to evaluate the role of Mig-6 in white adipose tissue (WAT) and its impact on systemic glucose homeostasis using *Mig-6* transgenic mice.

**Methods:**

Human visceral fat samples were obtained from four obese and three lean women undergoing hysterectomy. Adipocyte-specific *Mig-6* knock-in (*Mig-6*^AdKI^) mice were generated and maintained on either a high-fat diet (HFD) or normal chow diet (NCD). Glucose tolerance tests (GTT) and insulin tolerance tests (ITT) were performed. We conducted histological examinations to observe tissue morphology and used quantitative PCR to assess adipokine mRNA expression.

**Results:**

Mig-6 expression was significantly reduced in the adipose tissue of obese mice and humans. *Mig-6*^AdKI^ mice exhibited improved glucose tolerance and insulin sensitivity under both NCD and HFD conditions, without changes in body weight or fat mass. The improvement in glucose homeostasis under NCD conditions was particularly noteworthy. Increased adiponectin mRNA levels were observed in the WAT of *Mig-6*^AdKI^ mice. Meanwhile, histological analysis did not observe any changes in adipose tissue morphology that could explain the improvement in systemic glucose homeostasis, although there were tendencies towards increased adipocyte size and inflammation in HFD-fed *Mig-6*^AdKI^ mice.

**Conclusion:**

Adipose-specific overexpression of Mig-6 improves systemic glucose tolerance and insulin sensitivity, suggesting its potential as a target for both the treatment and prevention of diabetes. These findings provide a reference for further research targeting EGFR or Mig-6 in adipose tissue, highlighting the metabolic role of Mig-6 in glucose homeostasis.

## Introduction

Insulin resistance is a characteristic feature of type 2 diabetes mellitus (T2DM) and plays a key role in associated metabolic abnormalities. Adipose tissue, along with the liver and skeletal muscle, is a major organ related to insulin resistance. It is now well recognized that white adipose tissue (WAT) serves not only as an energy storage depot but also as a metabolically active endocrine organ. Adipose tissue plays a crucial role in the regulation of whole-body energy balance and glucose homeostasis [1,2].

Mitogen-inducible gene 6 (Mig-6; also known as Gene 33, ERRFI1, or RALT) is located in human chromosome 1p36. Mig-6 is a negative feedback regulator of receptor tyrosine kinase (RTKs) such as epidermal growth factor receptor (EGFR) [3]. Expression of Mig-6 is induced by many ligands of RTKs including EGF, hepatocyte growth factor (HGF), and fibroblast growth factor (FGF) via the mitogen-activated protein kinase (MAPK)/extracellular signal-regulated kinase (ERK) signaling pathway [4]. Mig-6 is induced rapidly upon EGFR activation, then negatively regulates EGFR signaling by two mechanisms: (1) inhibition of EGFR catalytic activation by docking onto EGFR kinase domain, (2) induction of endocytosis and degradation of EGFR/Mig-6 complex [5,6]. Previous studies have demonstrated that increased Mig-6 suppresses EGFR signaling in vitro assays [7,8] and Mig-6 deficient mice showed increased EGFR and EGFR downstream signaling [9,10]. The role of Mig-6 as a tumor suppressor in EGFR-dependent cancers is well understood [3], and recent studies have also demonstrated its involvement in glucose and lipid metabolisms. Liver specific deletion of *Mig-6* induced hepatic lipid accumulation and increased serum cholesterol levels [11,12]. Liver specific ablation of *Mig-6* also causes hyperglycemia and insulin resistance in mice by increasing phosphorylation of insulin receptor substrate 1 (IRS-1) at serine 307 via EGFR-dependent manner [13]. Another study demonstrated that Mig-6 is induced by endoplasmic reticulum (ER) stress and promotes glucolipotoxicity-induced pancreatic beta cell death [14]. *Mig-6* haploinsufficiency also showed a protective effect against streptozotocin-induced diabetes by increasing beta cell mass recovery [15]. These studies have shown that the effects of *Mig-6* are mediated through EGFR-dependent mechanisms. However, the metabolic roles of Mig-6 and EGFR in adipose tissue remain poorly understood. The findings to date are predominantly based on a limited number of in vitro studies. The aim of this study is to evaluate the role of Mig-6 in adipose tissue, with the ultimate goal of understanding the metabolic role of EGFR. We investigated whether modulating *Mig-6* in WAT affects systemic glucose tolerance and insulin sensitivity using *Mig-6* transgenic mice.

## Methods

### Human samples

Human adipose tissue samples were provided by the department of Obstetrics and Gynecology at the Chungnam National University hospital through collaboration. Human visceral fat samples were obtained from four obese (Body mass index, BMI≥25 kg/m^2^) and three lean women (BMI<25 kg/m^2^) undergoing hysterectomy. Body mass index was calculated as weight divided by squared height value.

### Animal models

Adipocyte specific *Mig-6* knock-in (*Mig-6*^AdKI^) mice were generated by mating *Mig-6* overexpression mice with Rosa26 locus [16] with Adiponectin-*Cre* mice (Jackson Laboratory, Stock No. *028020*, Bar Harbor, ME, USA) on a C57BL/6 background. All mice were maintained in a controlled environment (12 h light/12 h dark cycle; humidity 50–60%; ambient temperature 22 ± 2°C). Mice were allowed free access to tap water and feed. Mice were fed high fat diet (HFD; Research Diet, D12492) or normal chow diet (NCD) for 12 weeks from 6 weeks of age. Mice were euthanized with carbon dioxide delivered at a regulated flow rate followed by cervical dislocation. Energy expenditure and ambulatory activity were determined by indirect calorimetry [17]. InAnlyzer (MEDIKORS; Seongnam, Korea) was used to carry out dual energy X-ray absorptiometry (DXA) analysis.

### Glucose tolerance tests (GTT) and insulin tolerance tests (ITT)

GTT and ITT were performed at 18–19 weeks of age. For GTT, mice were fasted for 16 hours. NCD-fed mice received an intraperitoneal injection of glucose at a dose of 2g per kilogram of body weight, while HFD-fed mice received a dose of 1g per kilogram of body weight. Blood glucose levels were measured with a glucometer (AccuChek^®^) at 0, 15, 30, 60, 90, and 120 min. Mice were fasted for 6 h to the insulin tolerance test. Next, 0.75 U of insulin (Humalog^®^) per kilogram of body weight was injected into the intraperitoneal cavity and blood glucose levels were measured with a glucometer at 0, 15, 30, 60, 90, and 120 min.

### Histological analysis

All tissue samples were obtained from 21-week-old mice. After mice were sacrificed, tissue were collected, fixed, and embedded in paraffin. Hematoxylin and eosin (H&E) staining and immunohistochemical staining against Mig-6 were performed according to standard protocols. The smallest and largest diameters of each cell were measured on structurally distinct adipocytes using the Image J software. The mean of these two values was used in analyses [18].

### Biochemical measurements

Blood was collected from the mouse heart under general anesthesia. Samples were centrifuged at 5,000 rpm for 5 min and the supernatants were collected. Biochemical analyses, including determination of cholesterol and triglyceride levels, were performed using a Hitachi 7180 auto analyzer and Wako reagents (Wako Pure Chemical Industries, Ltd., Osaka, Japan).

### Western blot analysis

Tissues were lysed in RIPA buffer (30 mmol/L Tris, pH 7.5, 150 mmol/L sodium chloride, 1 mmol/L phenylmethylsulfonyl fluoride, 1 mmol/L sodium orthovanadate, 1% Nonidet P-40, 10% glycerol, and phosphatase and protease inhibitors). Western blot analysis of 30–50 μg protein was performed according to standard procedures using the following commercially available antibodies; Mig-6 (GTX32242; GeneTex, Irvine, CA, USA), β-actin (A2066; Sigma-Aldrich, St. Louis, MO, USA), GAPDH (Cell Signaling Technology, Danvers, MA, USA, 2118). Appropriate secondary antibodies were obtained from Santa Cruz Biotechnology for anti-rabbit, and Cell Signaling Technology for anti-mouse.

### RNA isolation and real-time PCR

Total RNA was extracted using TRIzol reagent (Thermo Fisher Scientific, Waltham, MA, USA), and complementary DNA (cDNA) was prepared using M-MLV reverse transcriptase and oligo-dT primers (Invitrogen, Carlsbad, CA, USA). The resultant cDNA was amplified using Rotor-Gene 6000 realtime rotary analysis software version 1.7 (Corbett Life Science, Sydney, Australia). Quantitative real–time PCR was performed using QuantiTect^TM^SYBR^®^Green PCR Master Mix (Qiagen, Hilden, Germany). The comparative Ct method (*ΔΔCT*) was used to quantify the trascripts, and values are expressed as -fold differences.

### Statistical analysis

All statistical analyses were performed using SPSS statistical software for Windows (version 21.0; SPSS Inc., Chicago, IL, USA) and GraphPad Prism software (version 8.0; GraphPad Software, Inc., San Diego, CA, USA). Data are reported as the mean ± standard error of the mean (SEM). Data of the four groups were analyzed using one-way analysis of variance (ANOVA) followed by Tukey’s honest significant difference post-hoc test, and a two-tailed Student’s t test was used to determine differences of the two groups. Body weight changes, GTT and ITT were analyzed using a repeated measure two-way ANOVA with both time and group as sources of variation. P value < 0.05 was considered to be statistically significant (*; p < 0.05, **; p < 0.01 and ***###; p < 0.001).

### Ethnical statement

Written informed consent was obtained from all participants who provided human adipose tissue samples. This study was approved by the Institutional Review Board of Chungnam National University Hospital (approval number: 2016-07-026) and conducted in accordance with the principles of the Declaration of Helsinki. All experimental procedures involving mice were conducted in accordance with the guidelines of the Institutional Animal Care and Use Committee of the Chungnam National University School of Medicine.

## Results

### 1. Obesity is associated with a reduction of Mig-6 levels in adipose tissue from both mice and humans

**Fig 1:**
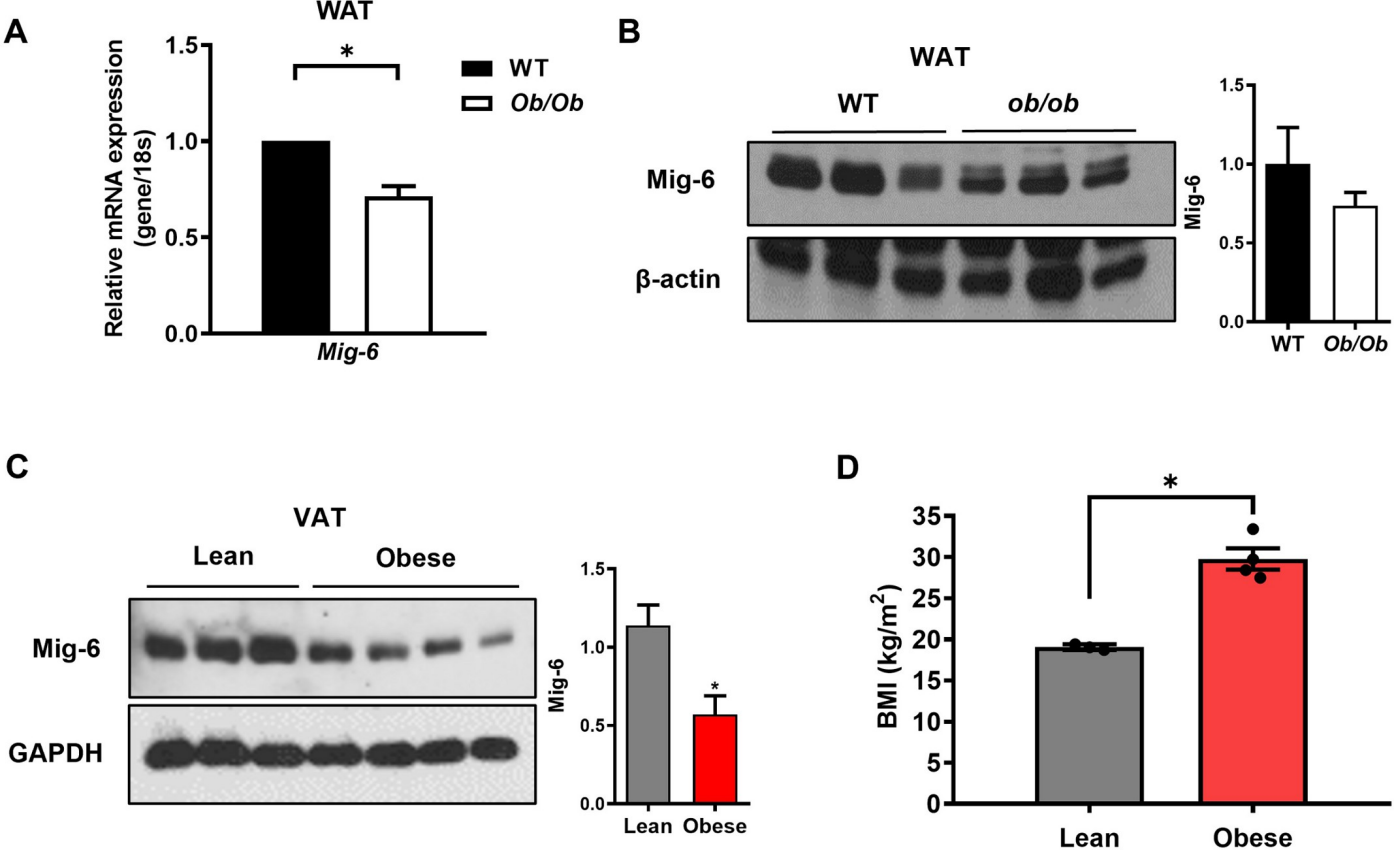
Expression of Mig-6 in adipose tissue from obese mice and humans. **(A**) Mig-6 mRNA levels and **(B)** protein levels in WAT from wild type (WT) and *ob/ob* mice (n = 2–3 mice/group). **(C)** Protein levels of Mig-6 in visceral adipose tissue from lean (n = 3) and obese (n = 4) subjects. **(D)** Body mass index of lean (19.1 ± 0.2 kg/m^2^) and obese (29.8 ± 1.3 kg/m^2^) subjects. Data are presented as means ± SEM. *P < 0.05 versus controls.

To determine whether Mig-6 expression is altered in obesity, we examined *Mig-6* mRNA levels in adipose tissue from wild type (WT) and *ob/ob* mice. As shown in Fig 1A and 1B, a significant reduction in *Mig-6* mRNA and protein expression was observed in the WAT from the *ob/ob* mice. We also examined Mig-6 expression in fat samples from four obese and three lean human individuals. The mean BMI was 19.1 ± 0.2 kg/m² for lean participants and 29.8 ± 1.3 kg/m² for obese participants. Clinical characteristics of the participants are detailed in S1 Table. Mig-6 expression was significantly decreased in the visceral adipose tissue (VAT) of obese subjects compared to that of lean subjects (Fig 1C). This suggested that Mig-6 expression is dysregulated in the obese adipose tissue of both mice and humans.

### 2. Generation of adipose-specific *Mig-6* knock-in mice

**Fig 2:**
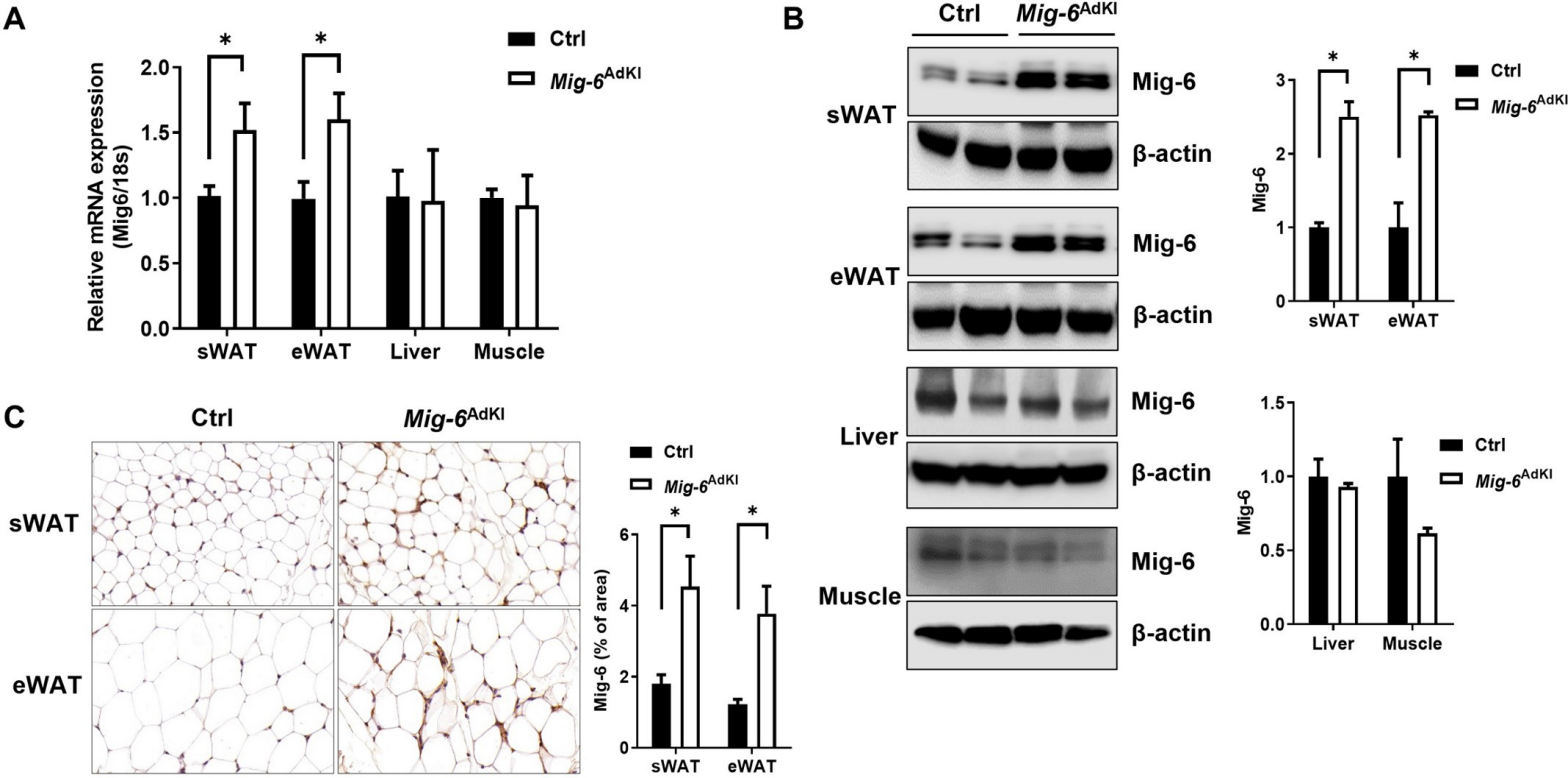
Generation of adipose specific *Mig-6* knock-in mice. **(A)** The mRNA expression and **(B)** protein levels of Mig-6 in sWAT, eWAT, liver and muscle of 21-week-old control and *Mig-6*^AdKI^ mice (n = 2–4 mice/group). **(C)** Immunohistochemical staining of Mig-6 in WAT from control and *Mig-6*^AdKI^ mice. Data are presented as mean ± SEM. *P < 0.05 versus controls.

We generated adipocyte-specific *Mig-6* knock-in mice on the C57BL/6 background. *Mig-6*^AdKI^ mice were obtained by breeding *Mig-6* overexpression mice with Rosa26 locus with Adiponectin-*Cre* mice. *Mig-6*^AdKI^ mice showed increased *Mig-6* mRNA and protein expression in subcutaneous and epididymal WAT (sWAT and eWAT). However, Mig-6 expression was unaffected in other insulin-sensitive tissues such as the liver and muscle (Fig 2A and 2B). Immunohistochemical staining for Mig-6 also revealed increased Mig-6 expression in the WAT from *Mig-6*^AdKI^ mice (Fig 2C).

### 3. *Mig-6*^AdKI^ mice improve glucose metabolism without altering body weight

**Fig 3:**
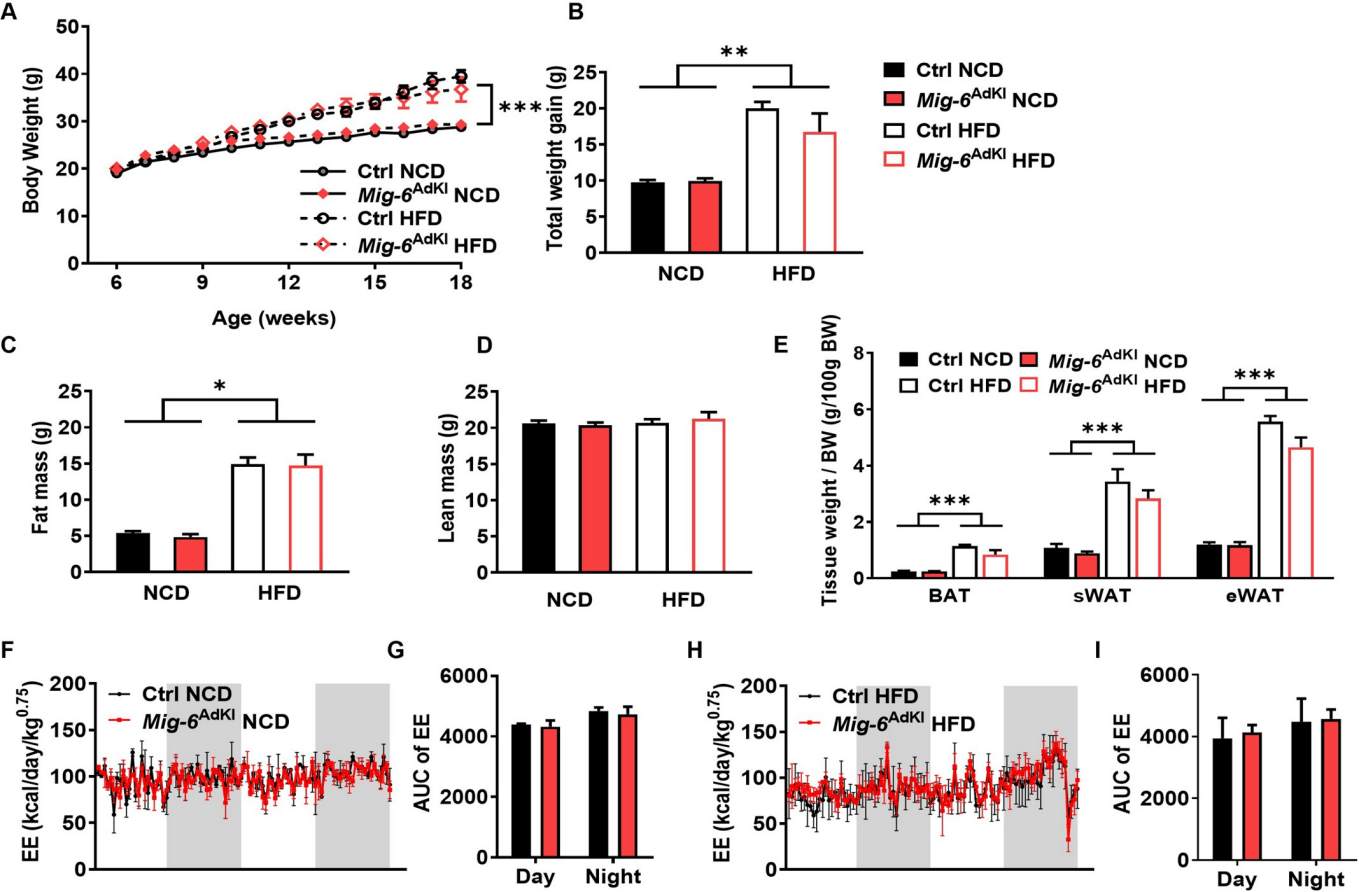
Body weight, fat mass, and energy expenditure in *Mig-6*^AdKI^ mice. **(A, B)** Body weight gain of control and *Mig-6*^AdKI^ mice fed either a normal chow diet (NCD) or a high fat diet (HFD) for 12 weeks starting at 6 weeks of age (n = 4-9/group). **(C, D)** Fat and lean mass of control and *Mig-6*^AdKI^ mice as measured by DXA after 12 weeks on diet (n = 4-9/group). **(E)** Tissue weight of each adipose depot (BAT, sWAT and eWAT) (n = 4-9/group)**. (F-I)** Energy expenditure in control and *Mig-6*^AdKI^ mice (n = 2-3/group). Data are mean ± SEM. *P < 0.05, **p < 0.01, ***p < 0.001.

The mice were initially fed a normal chow diet (NCD), and then some were switched to a high fat diet (HFD) at 6 weeks of age. The weight of the animals in each group was measured weekly. Weight gain was significantly greater between 6 and 18 weeks of age in mice fed on HFD than in those fed on NCD. However, there was no statistically significant effect of *Mig-6* overexpression on absolute body weight or body weight gain (Fig 3A and 3B) nor on body composition as measured by DXA (Fig 3C and 3D). Additionally, the tissue weight of each adipose tissue depot showed no significant difference between the groups (Fig 3E). There were no differences in energy expenditure (Fig 3F–3I).

**Fig 4:**
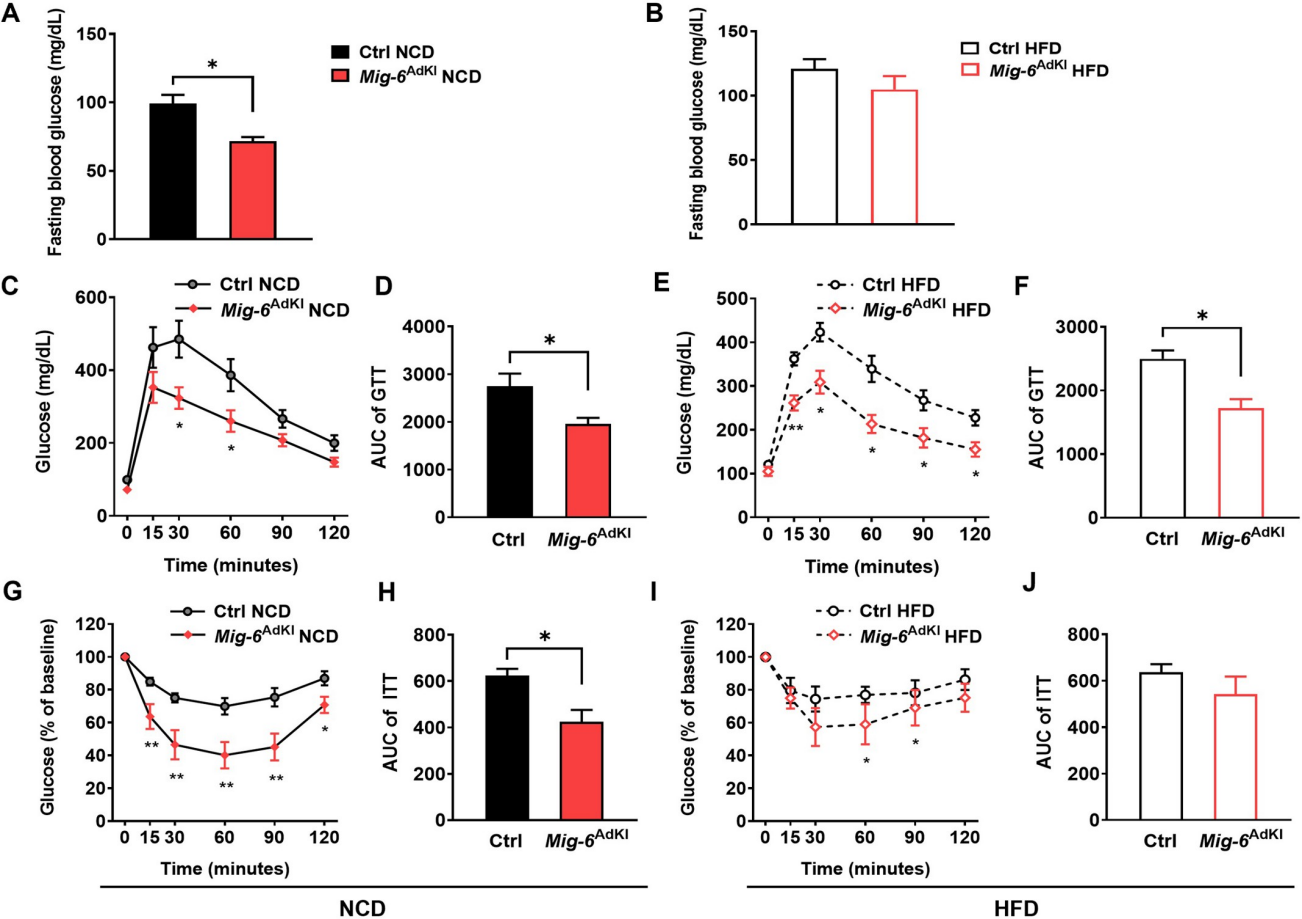
*Mig-6*^AdKI^ mice exhibit improved glucose tolerance and insulin sensitivity under both NCD and HFD conditions. **(A, B)** Fasting blood glucose levels in control and *Mig-6*^AdKI^ mice fed either a normal chow diet (NCD) or a high fat diet (HFD) (n = 4-8/group). **(C-F)** Glucose tolerance tests (GTT) were performed on fasted control and *Mig-6*^AdKI^ mice after 12 weeks of NCD or HFD feeding. Blood glucose concentrations were measured after glucose injection (2g/kg for NCD-fed mice and 1g/kg for HFD-fed mice), and area under the curves were calculated (n = 4-8/group). **(G-J)** Insulin tolerance tests were performed on fasted control and *Mig-6*^AdKI^ mice fed either a normal chow diet (NCD) or a high fat diet (HFD) (n = 4-8/group). Mice were injected with insulin (0.75 U/Kg) and blood glucose concentration were measured over 90 min. Points represent glucose levels (mean ± SEM) at each respective time point. Bar graphs represent area under the curve (mean ± SEM) for all time points. *P < 0.05, **p < 0.01.

Systemic glucose homeostasis was assessed in *Mig-6*^AdKI^ mice. Fasting blood glucose levels were significantly decreased in *Mig-6*^AdKI^ mice compared to control mice under NCD condition, but this difference was not significant in mice fed a HFD (Fig 4A and 4B). To further investigate systemic glucose tolerance and insulin sensitivity, we performed GTT and ITT on *Mig-6*^AdKI^ mice. Mice fed a HFD showed significantly increased insulin resistance, whereas *Mig-6*^AdKI^ were protected from HFD-induced glucose intolerance and insulin resistance. Moreover, improved glucose tolerance and insulin sensitivity were also observed in *Mig-6*^AdKI^ mice fed on NCD (Fig 4C–4J).

### 4. Histological analysis of WAT in *Mig-6*^AdKI^ mice

**Fig 5:**
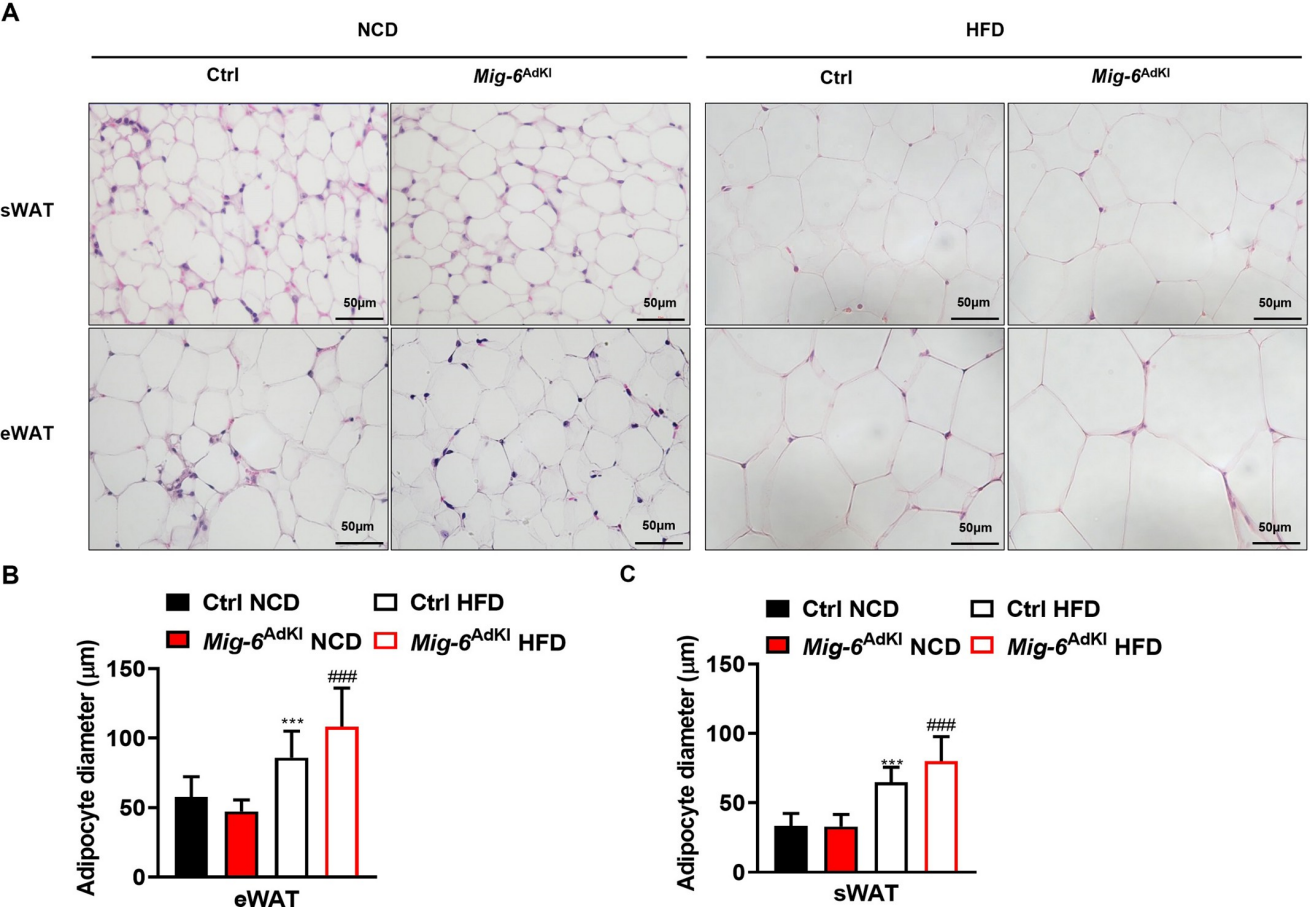
Histological analysis of WAT from control and *Mig-6*^AdKI^ mice. **(A)** Hematoxylin-eosin (H&E) staining was performed on sWAT and eWAT from 21- week-old control and *Mig-6*^AdKI^ mice. **(B, C)** The diameter of adipocytes was measured using Image J software. Data are presented as mean ± SEM. ***P < 0.001 versus control mice fed a normal chow diet (NCD), ###*P* < 0.001 versus control mice fed a high fat diet (HFD).

Adipocyte hypertrophy and adipose tissue inflammation are closely associated with the development of systemic insulin resistance. When fat cells undergo hypertrophy, it leads to adipose tissue dysfunction and triggers a pro-inflammatory environment. Therefore, we performed H&E staining to evaluate the histological characteristics. There was no significant difference in adipose tissue morphology between *Mig-6*^AdKI^ and WT mice fed a NCD. However, H&E staining of WAT revealed an increase in adipocyte size in *Mig-6*^AdKI^ mice under a HFD condition (Fig 5A–5C).

### 5. Alteration in gene expression of adipokines in *Mig-6*^AdKI^ mice

**Fig 6:**
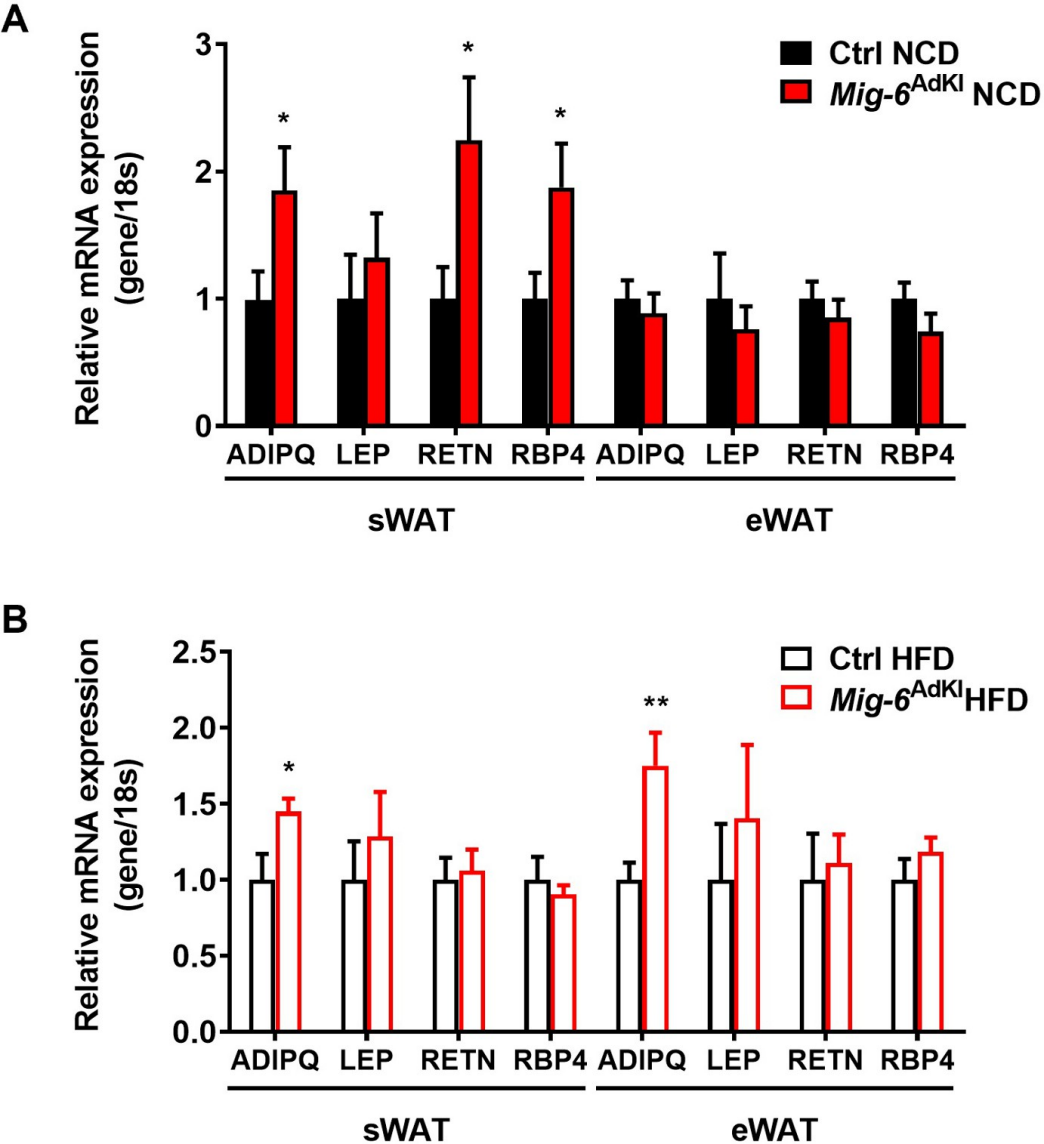
Expression of adipokine genes in WAT from control and *Mig-6*^AdKI^ mice. **(A, B)** The mRNA expression of ADIPQ (adiponectin), LEP (leptin), RETN (resistin), RBP4 (retinol binding protein 4) in sWAT and eWAT from 21- week-old control and *Mig-6*^AdKI^ mice**. (A)** Mice were fed a normal chow diet (NCD) (n = 4–7 per group). **(B)** Mice were fed a high-fat diet (HFD) (n = 7–8 per group). The mRNA levels were normalized relative to 18S, and the level in control mice is set as 1. Data are presented as mean ± SEM. *P < 0.05, **p < 0.01.

We further investigated the expression levels of adipokine genes, which are associated with insulin sensitivity, in both sWAT and eWAT. Increased levels of adiponectin mRNA were observed in sWAT of NCD-fed mice, even in those given a HFD. In HFD-fed mice, we observed an increase in adiponectin mRNA levels in eWAT as well. In NCD-fed *Mig-6*^AdKI^ mice, there were changes in the levels of other adipokines, such as resistin and retinol-binding protein 4 (RBP4) in addition to adiponectin (Fig 6A and 6B).

## Discussion

In this study, we have provided the first evidence for the metabolic role of Mig-6 in adipose tissues. We found that Mig-6 levels are reduced in adipose tissue from obese individuals and *ob/ob* mice with hyperinsulinemia, despite stress and insulin being strong inducers of Mig-6 expression. Although *Mig-6* is well-known immediate early response gene that is rapidly induced by various stimuli, this study suggests that *Mig-6* is significantly decreased in chronic stress conditions such as obesity.

We also demonstrated that adipose-specific *Mig-6* overexpression improves systemic glucose tolerance and insulin sensitivity. Remarkably, these metabolic benefits were observed in *Mig-6*^AdKI^ mice under both HFD and NCD conditions. The improvements in glucose homeostasis observed under NCD conditions are particularly noteworthy. These findings suggest that Mig-6 has the potential to be a target for diabetes prevention. Given the continuous global rise in the prevalence of T2DM, the substantial socioeconomic costs, and the increased risk of complications even prior to diagnosis, diabetes prevention possesses significant clinical importance. Furthermore, T2DM is a heterogeneous metabolic disorder characterized by complex pathogenesis, and it is well recognized that not all individuals with T2DM are overweight or obese. Therefore, the improvement in glucose homeostasis observed under NCD conditions indicates that targeting adipose Mig-6 could be beneficial not only for diabetes prevention but also as a therapeutic target for non-obese diabetic individuals.

In this study, we utilized transgenic mice in which *Mig-6* was overexpressed in adipocytes to ultimately investigate the metabolic role of EGFR. Because Mig-6 serves as an endogenous negative feedback inhibitor of EGFR, it is a suitable target for regulation of EGFR signaling. We have previously demonstrated that activity of EGFR signaling is inversely correlated with Mig-6 expression in transgenic mice with *Mig-6* [10]. In liver-specific *Mig-6* ablation mice, insulin resistance increased in an EGFR-dependent manner [13]. Conversely, EGFR inhibition decreased liver de novo lipogenesis, leading to improvements in HFD-induced hepatic steatosis and glucose intolerance [19]. Nonetheless, the potential for an EGFR-independent role of Mig-6 cannot be completely ruled out, and further studies are required to explore this possibility.

*Mig-6*^AdKI^ mice exhibited improvements in glucose metabolism without altering total body weight. As the first potential mechanism, we considered the possibility that, despite no changes in total body weight, there were alterations in adipose tissue characteristics. Previous studies have reported that, although EGFR has an inhibitory effect on preadipocyte differentiation, it enhanced lipogenic gene expression and promoted adipogenesis in differentiated adipocytes in vitro [20,21]. However, in this study, no histological changes, such as a decrease in adipocyte size or inflammation, were observed in WAT that could explain the improvement in insulin sensitivity. In NCD-fed *Mig-6*^AdKI^ mice, there was a slight trend toward smaller adipocyte size, but this difference was not statistically significant. Instead, contrary to our expectations, HFD-fed *Mig-6*^AdKI^ mice exhibited an increase in adipocyte size in WAT. Although we cannot determine a definitive cause for the differences in adipose tissue histology between the NCD and HFD groups, two hypotheses can be considered. First, EGFR signaling may result in diverse consequences across various metabolic pathways. Inhibition of EGFR signaling may promote HFD-induced TG accumulation. Second, we cannot rule out the possibility that Mig-6 plays a minimal or different role in chronic stress conditions. The disparity between NCD and HFD mice may have influenced the observed results, which showed a more significant improvement in glucose homeostasis among the NCD mice.

As a second potential mechanism for the improvement in glucose metabolism without changes in body weight, we considered the possibility of alterations in adipokine levels. Adipose tissue is well recognized as an endocrine organ and produces various adipokines, which communicate with other tissues to regulate systemic glucose homeostasis. Given the improved glucose homeostasis observed in *Mig-6*^AdKI^ mice without beneficial alterations in adipose tissue morphology, we investigated the potential effects of adipose-specific *Mig-6* overexpression on adipokine profiles. We demonstrated that adiponectin mRNA levels are elevated in the WAT of *Mig-6*^AdKI^ mice under both NCD and HFD conditions. Adiponectin, abundantly secreted by adipose tissue, acts on a diverse range of target organs and improves insulin sensitivity through various effects [22]. Decreased adiponectin secretion in obesity is a key factor contributing to the development of insulin resistance [23]. It has been suggested that the main mechanism by which adiponectin impacts insulin sensitivity is the suppression of hepatic glucose production [24,25]. Other mechanisms include increasing fatty acid oxidation and inhibiting inflammation in insulin-sensitive organs such as the liver and skeletal muscle. In this study, we focused exclusively on adipose tissue and examined only the changes in WAT. However, further research is necessary to investigate the potential changes in the liver and muscle of *Mig-6*^AdKI^ mice. Additionally, the molecular mechanism linking EGFR signaling or Mig-6 and adipokine secretion is not well understood. However, previous study reported that EGFR-TKI treatment increases the serum adiponectin levels in subjects with non-small cell lung cancer [26], but little is known about the molecular mechanism linking EGFR signaling or Mig-6 and adipokine secretion. In several studies, inhibition of EGFR signaling has been demonstrated to diminish the activity of the phosphatidylinositol 3-kinase (PI3K)/Akt pathway, thereby reducing the mammalian target of rapamycin (mTOR) activity [27,28]. This reduction can indirectly lead to the activation of the AMP-activated protein kinase (AMPK). Given that the AMPK pathway plays a pivotal role in the secretion of adiponectin, the crosstalk between the EGFR and AMPK pathways may have contributed to the observed increase in adiponectin levels in our study. However, further investigation is required to thoroughly elucidate this potential mechanism.

This study has several limitations. Firstly, it remains unclear whether the observed improvements in systemic glucose tolerance and insulin sensitivity in *Mig-6*^AdKI^ mice are primarily due to EGFR inhibition in adipose tissue or if they result from the EGFR-independent metabolic roles of Mig-6. Secondly, although we hypothesize that increased adiponectin levels contributed to the enhanced systemic glucose homeostasis in *Mig-6*^AdKI^ mice, we did not assess the related changes in glucose metabolism in the liver and skeletal muscle. Furthermore, it is necessary to confirm whether circulating adiponectin levels actually increased. Future studies should aim to elucidate the precise mechanisms underlying the improvement in systemic glucose homeostasis and determine why the improvement in systemic glucose homeostasis is less pronounced under HFD conditions compared to NCD conditions.

## Conclusion

In conclusion, we demonstrated that adipose-specific *Mig-6* overexpression improves systemic glucose tolerance without altering body weight or fat mass in both NCD- and HFD-fed mice. Moreover, *Mig-6*^AdKI^ mice showed decreased fasting glucose and improved insulin sensitivity in NCD group. We also found that expression of adiponectin is significantly increased in WAT of *Mig-6*^AdKI^ mice. It is suggested that the increase in adiponectin is involved in the improvement of systemic glucose homeostasis, but further investigation into the relevant mechanisms is needed in the future. To our knowledge, this is the first study to confirm the metabolic role of Mig-6 in adipose tissue. These findings provide a reference for further research targeting EGFR or Mig-6 in adipose tissue. Additionally, these results suggest that adipose Mig-6 has potential as a target for both the treatment and prevention of diabetes.

## Supporting information

S1 Table Clinical characteristics of the participants. (DOCX)

S1 File Raw images. (PDF)

S2 File Raw data. (XLSX)
