# Supplementary material for: Role of Mig-6 in adipose tissue: Implications for glucose metabolism and insulin resistance
Source: PLoS One. 2025 Feb 12;20(2):e0314289. doi: 10.1371/journal.pone.0314289 (PMC11819470; doi:10.1371/journal.pone.0314289)
Supplement: S1 Table — (DOCX) [file pone.0314289.s001.docx]

**Table S1. Clinical characteristics of the participants**

|  | **Lean (n=3)** | **Obese (n=4)** | **p-value** |
| --- | --- | --- | --- |
| Age (years) | 40.7±8.1 | 38.8±6.3 | 0.858 |
| Height (cm) | 159.5±3.6 | 160.7±7.9 | 0.724 |
| Weight (kg) | 48.5±2.7 | 77.1±10.8 | 0.034 |
| BMI (kg/m^2^) | 19.1±0.4 | 29.8±2.6 | 0.034 |
| WC (cm) | 77.6±2.0 | 97.1±9.0 | 0.064 |
| FPG (mg/dL) | 83.3±4.6 | 90.5±0.6 | 0.029 |
| Fasting insulin (μIU/mL) | 5.9±2.0 | 9.8±4.3 | 0.289 |
| Fasting C-peptide (ng/mL) | 0.7±0.5 | 1.2±0.9 | 0.480 |
| HOMA-IR | 1.2±0.4 | 2.2±1.0 | 0.157 |
| Triglycerides (mg/dL) | 52.3±28.7 | 165±125.1 | 0.157 |
| LDL-C (mg/dL) | 102.3±18.8 | 114.8±41.1 | 0.724 |
| HDL-C (mg/dL) | 65.3±14.3 | 49±17.3 | 0.154 |

Data are expressed as the means ± SD. BMI, Body Mass Index; WC, Waist Circumference; FPG, Fasting Plasma Glucose; HOMA-IR, Homeostatic Model Assessment of Insulin Resistance; LDL-C, Low-Density Lipoprotein Cholesterol; HDL-C, High-Density Lipoprotein Cholesterol
