# Supplementary material for: Role of Mig-6 in adipose tissue: Implications for glucose metabolism and insulin resistance
Source: PLoS One. 2025 Feb 12;20(2):e0314289. doi: 10.1371/journal.pone.0314289 (PMC11819470; doi:10.1371/journal.pone.0314289)
Supplement: S1 File — (PDF) [file pone.0314289.s002.pdf]

Raw Data Images:

Western bolt

Sample : mice WAT (WT, *ob/ob*)

Of Figure 1-B in the main text ; Western blot from figure 1-B-a, figure 1-B-b were imaged as a full-length western blot in the Mig6 (53kDa) and ß-actin (45kDa).

Figure 1.

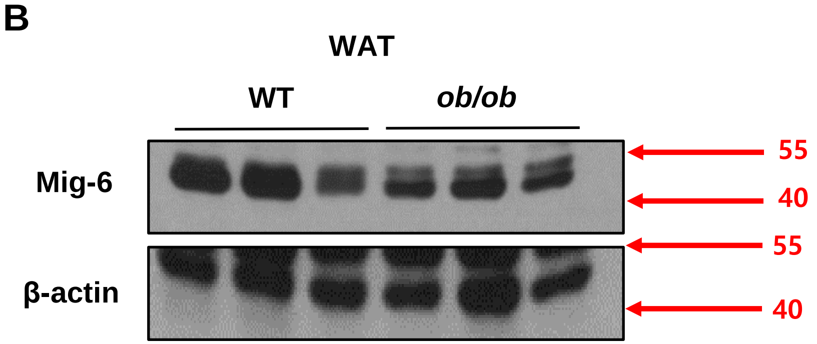

figure 1-B-a

protein; 10ug
antibody ; Mig6 ; #sc-137154 ; 53kDa

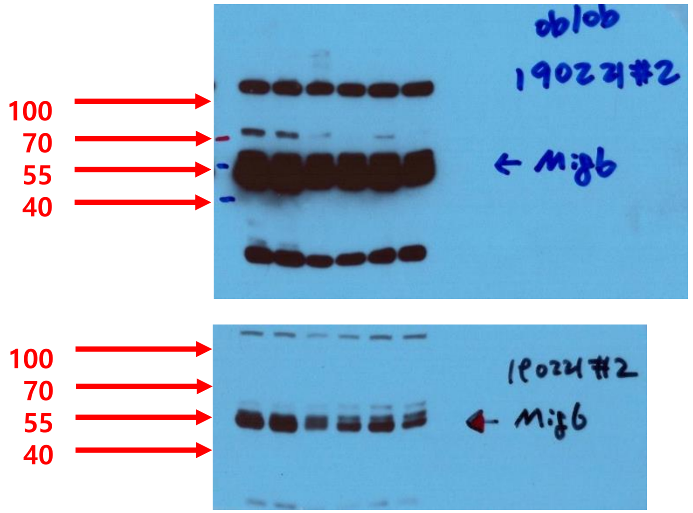

figure 1-B-b

protein; 10ug
antibody ; ß-actin ; #3700s ; 45kDa

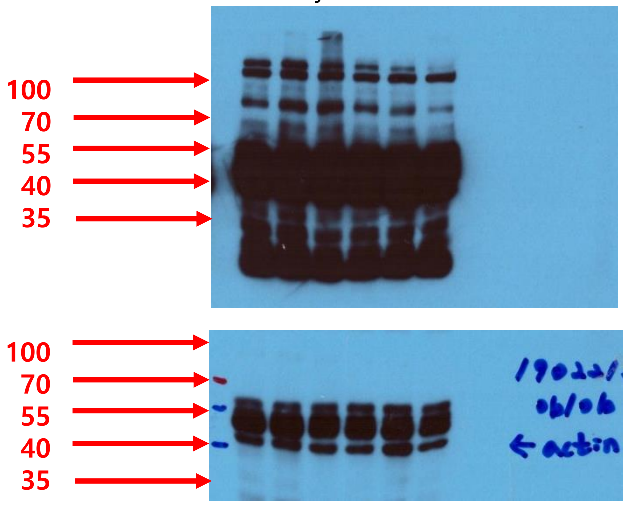

Figure 1.

C

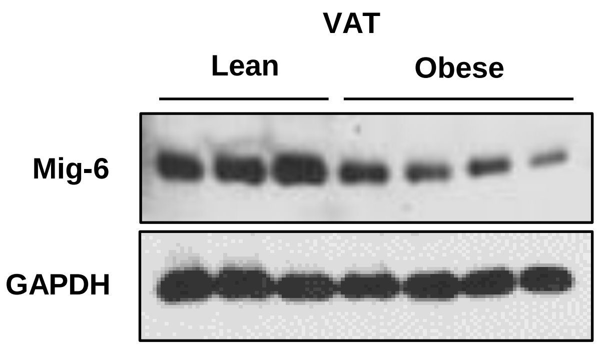
VAT
Lean
Obese
Mig-6
GAPDH

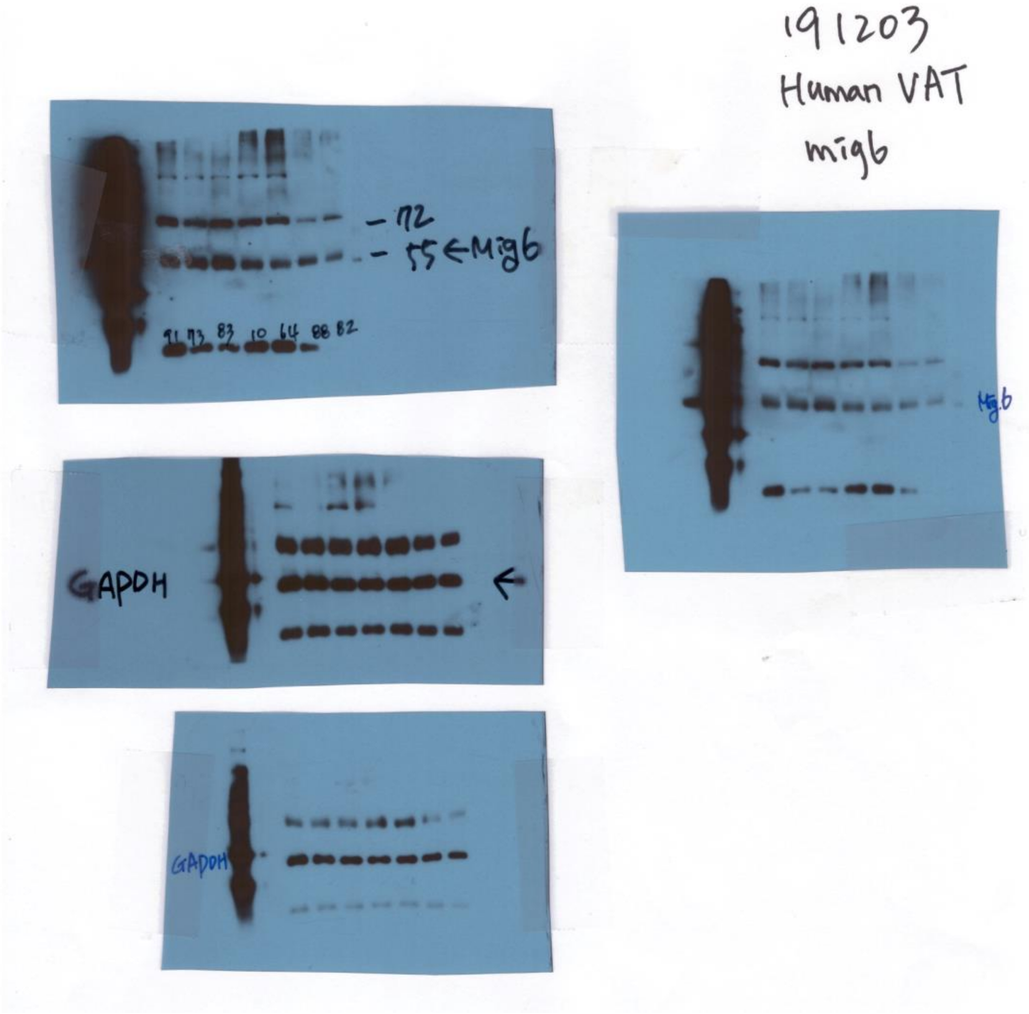
191203
Human VAT
mig6
72
55 ← Mig6
GAPDH
Mig6
GAPDH

Figure 1.

C

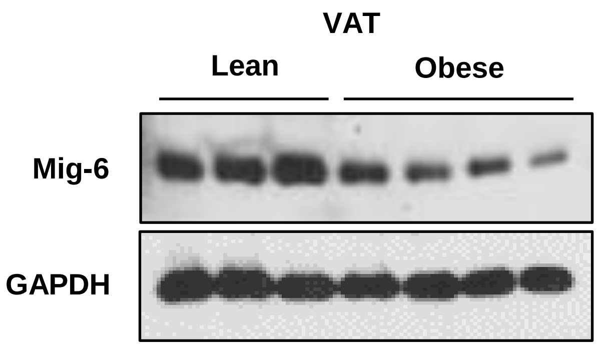
VAT
Lean
Obese
Mig-6
GAPDH

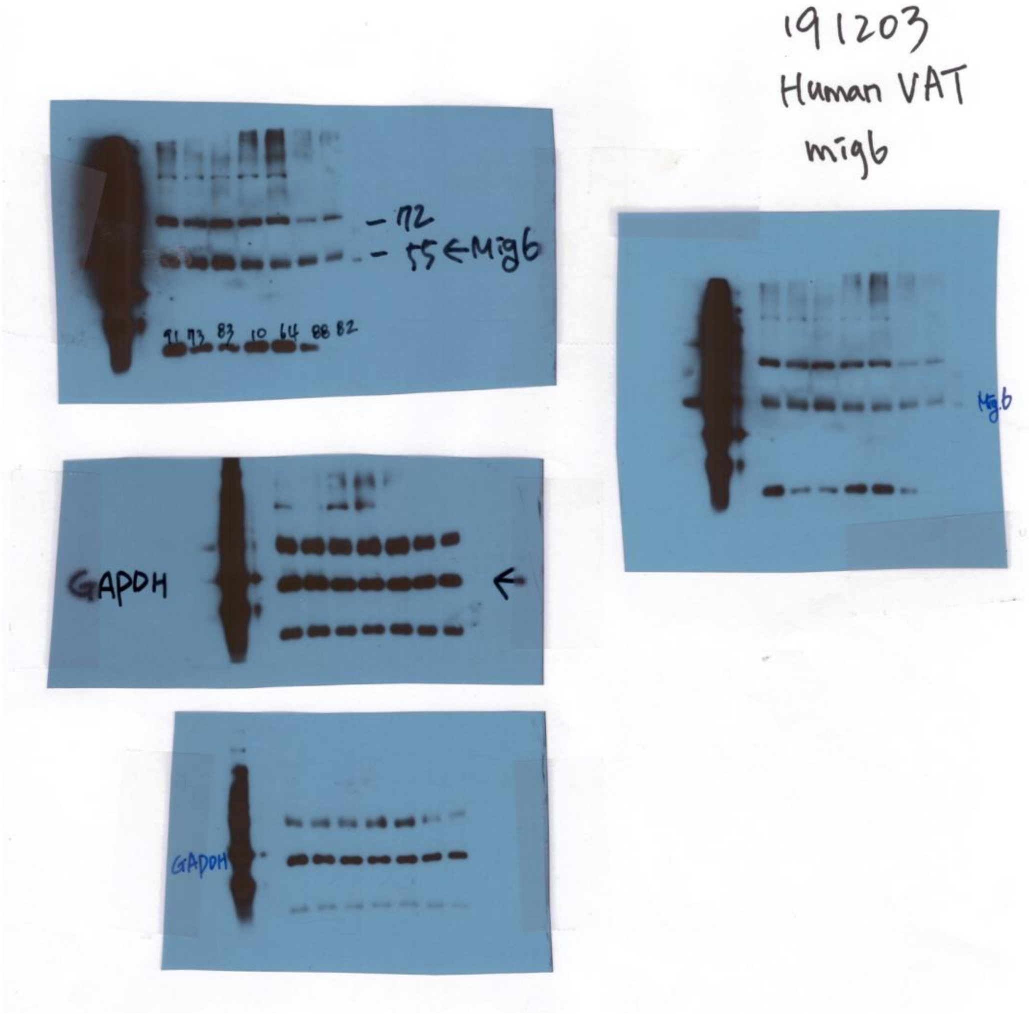
191203
Human VAT
mig6
72
55 ← Mig6
GAPDH
Mig6
GAPDH

Raw Data Images:

Western bolt

Sample : mice sWAT, eWAT (Ctrl, Mig6-ADKI)

Of Figure 2-B in the main text ; Western blot from figure 2-B-a was imaged as a full-length western blot in the Mig6 (53kDa). However, a non-specific reaction occurred(about 130~70kDa, 26kDa). Each western blot was cut prior to antibody hybridization above the 100kDa and 40kDa marker and imaged again. Mig6 signal was found to be improved when full-length Mig6 sections were removed (figure 2-B-b, figure 2-B-c). Each western blot was cut prior to antibody hybridization where each ß-actin (45kDa) section is from a single blot (figure 2-B-d, figure 2-B-e).

figure 2-B-a

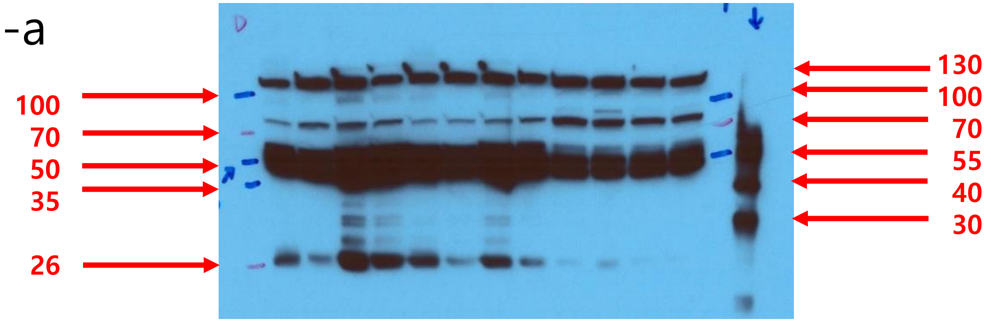

figure 2-B-b

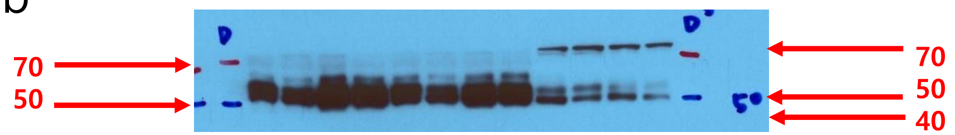

figure 2-B-c

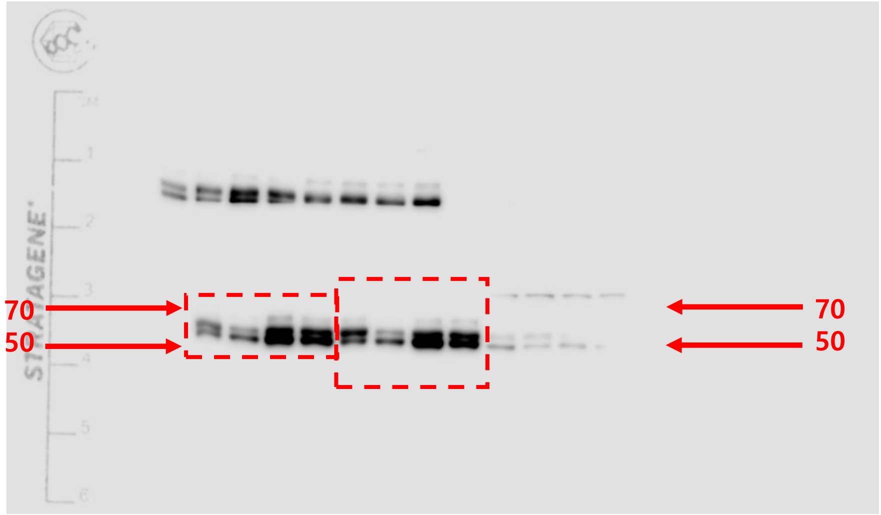

protein; 50ug
antibody ; Mig6 ; #sc-137154 ; 53kDa

figure 2-B-d

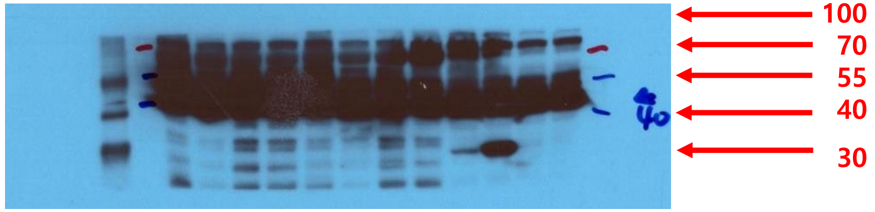

figure 2-B-e

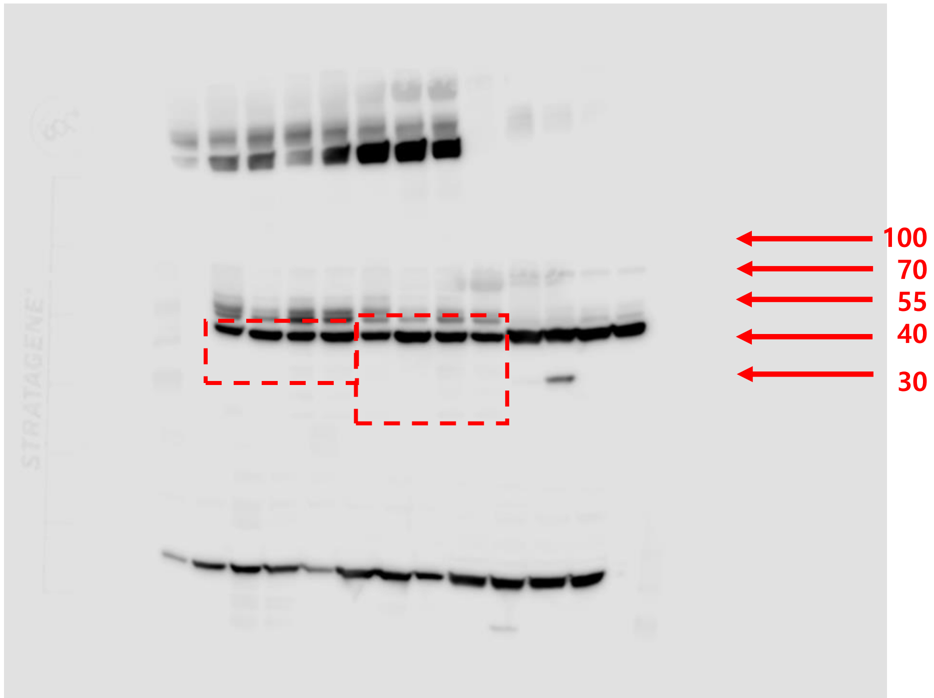

protein; 50ug
antibody ; ß-actin ; #3700s ; 45kDa

Raw Data Images:

Western bolt

Sample : mice Muscle (Ctrl, Mig6-ADKI)

Of Figure 2-B in the main text ; Western blot from figure 2-B-f was imaged as a full-length western blot in the Mig6 (53kDa). However, a non-specific reaction occurred(about 130~70kDa, 26kDa). Each western blot was cut prior to antibody hybridization above the 100kDa and 40kDa marker and imaged again. Mig6 signal was found to be improved when full-length Mig6 sections were removed (figure 2-B-g, figure 2-B-h). Each western blot was cut prior to antibody hybridization where each ß-actin (45kDa) section is from a single blot (figure 2-B-i, figure 2-B-j).

figure 2-B-f

figure 2-B-g

figure 2-B-h

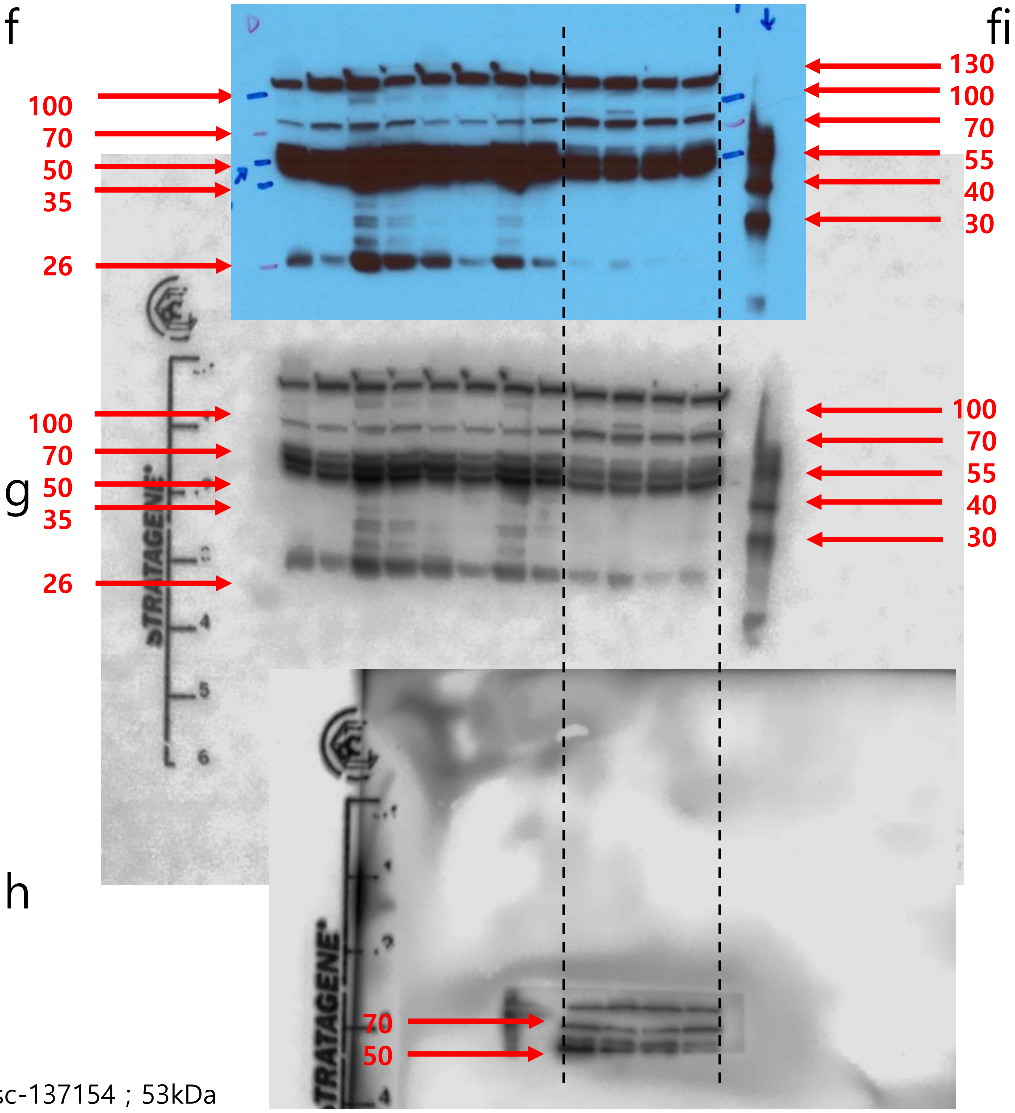

protein; 30ug
antibody ; Mig6 ; #sc-137154 ; 53kDa

figure 2-B-i

figure 2-B-j

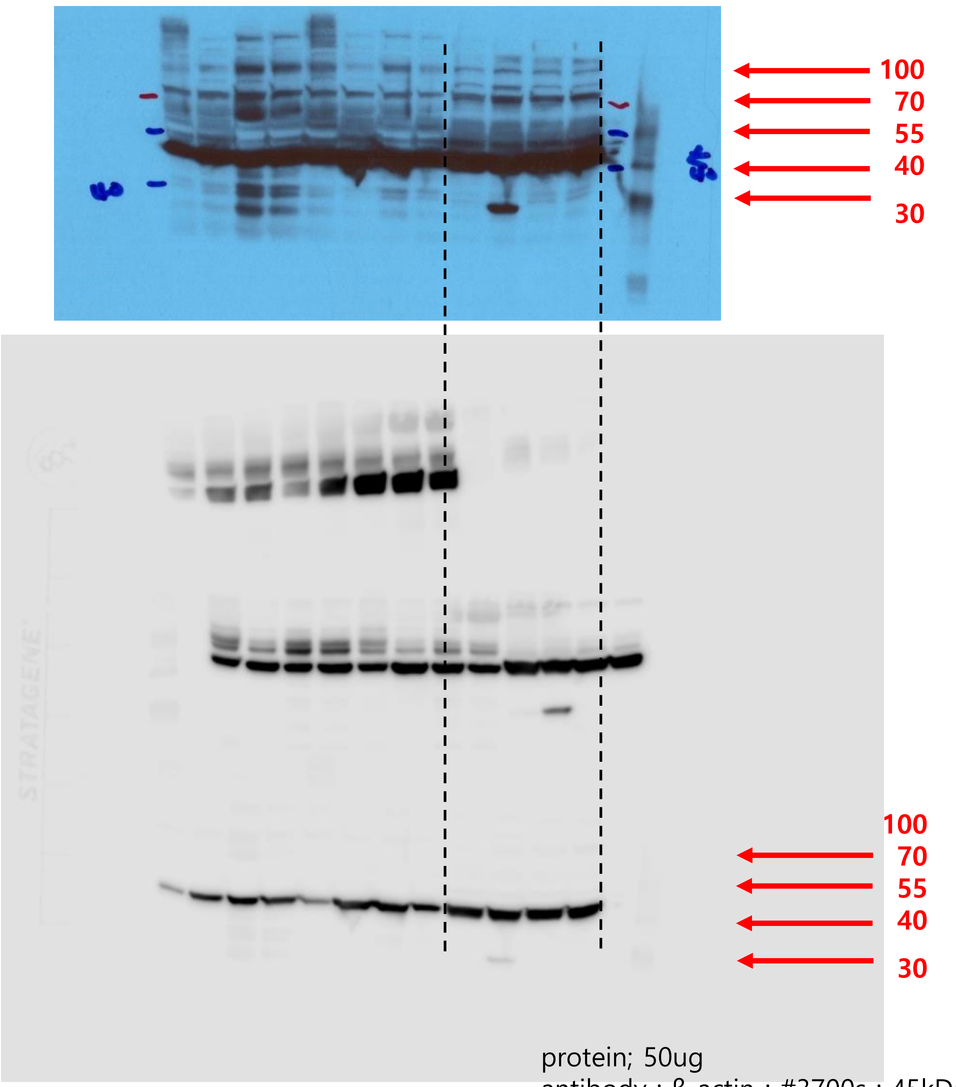

protein; 50ug
antibody ; ß-actin ; #3700s ; 45kDa

Raw Data Images:

Western bolt

Sample : mice Liver (Ctrl, Mig6-ADKI)

Of Figure 2-B in the main text ; Western blot from figure 2-B-k was imaged as a full-length western blot in the Mig6 (53kDa). However, a non-specific reaction occurred(about 130~70kDa, 26kDa). Each western blot was cut prior to antibody hybridization above the 100kDa and 40kDa marker and imaged again. Mig6 signal was found to be improved when full-length Mig6 sections were removed (figure 2-B-l, figure 2-B-m). Each western blot was cut prior to antibody hybridization where each ß-actin (45kDa) section is from a single blot (figure 2-B-n, figure 2-B-o).

figure 2-B-k

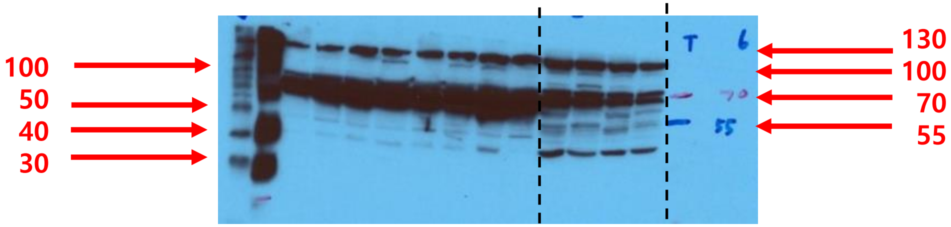

protein; 50ug
antibody ; Mig6 ; #sc-137154 ; 53kDa

figure 2-B-l

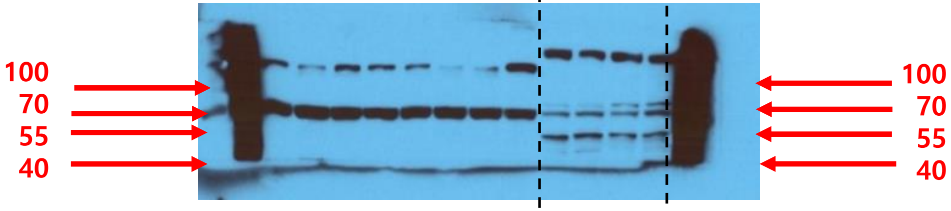

figure 2-B-m

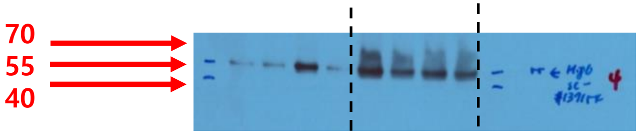

protein; 10ug
antibody ; Mig6 ; #sc-137154 ; 53kDa

figure 2-B-n

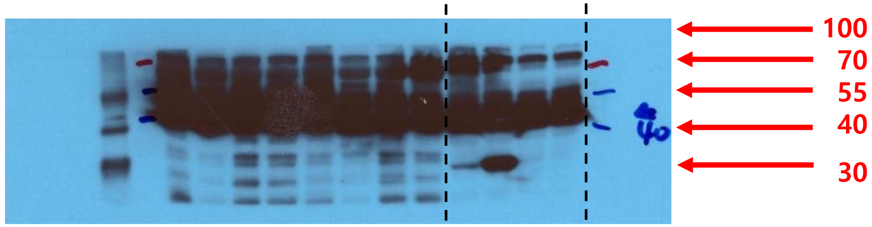

figure 2-B-o

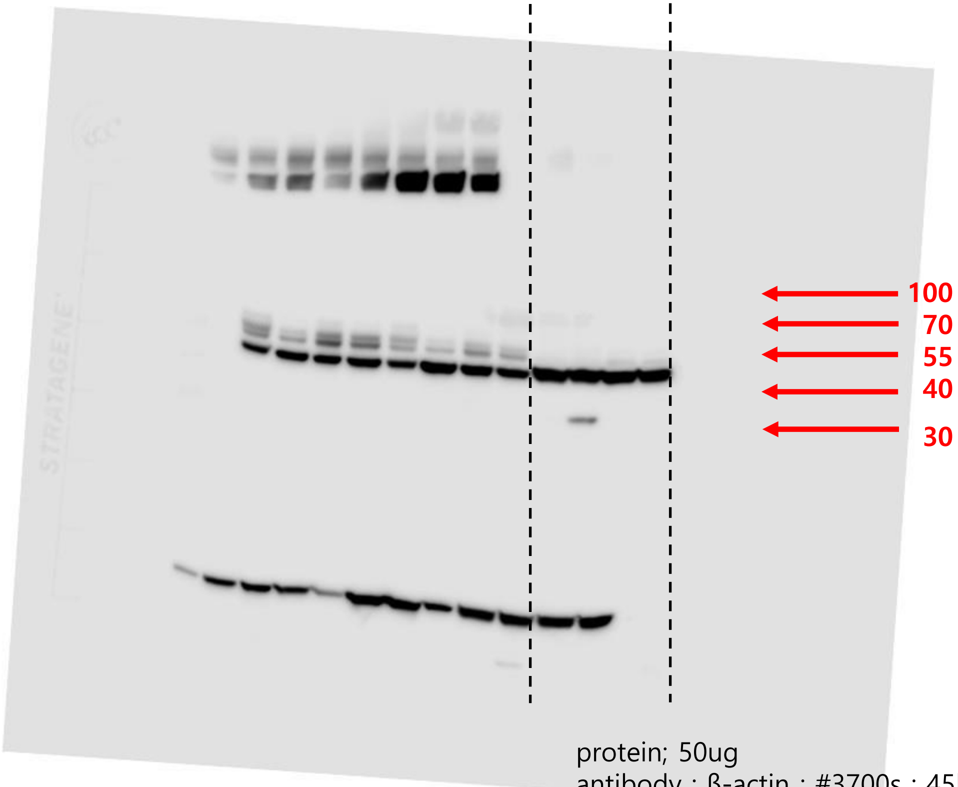

protein; 50ug
antibody ; ß-actin ; #3700s ; 45kDa
